# A Holistic Approach to Cardiopulmonary Concerns in a Schizophrenic Patient and Ensuring Quality Care

**DOI:** 10.7759/cureus.37303

**Published:** 2023-04-08

**Authors:** Rostam Vohoumani, Raymond Le, Davin Agustines

**Affiliations:** 1 Osteopathic Medicine of the Pacific, Western University of Health Sciences, Pomona, USA; 2 Psychiatry, Olive View - University of California Los Angeles Medical Center, Los Angeles, USA

**Keywords:** cardiopulmonary symptoms, patient-centered, schizophrenia, quality care, holistic care

## Abstract

“Holistic approach” and “Quality Care” have been trending topics in the medical community for several decades, with holistic practice advocating for meeting patients' emotional, physiologic, psychological, and spiritual needs. In comparison, the quality care model highlights patient care metrics of equity, safety, and timeliness in medical treatment. Both converge on the concept of medical care centered around patients, with countless papers discussing their benefits in various healthcare settings. However, there is a gap in the literature regarding using both models to manage the mentally ill. Adopting the healthcare quality approach and incorporating a holistic perspective in managing psychiatric conditions can improve patient satisfaction and reduce societal and healthcare costs, making it an important area of further investigation. This paper will discuss the case of a 59-year-old male with schizophrenia who developed cardiopulmonary symptoms during his prolonged hospital stay, leading to extensive yet inefficient workup, in which his symptoms improved after implementing measures that uplifted his psyche. It will carefully examine holistic care's emotional and psychological aspects and the quality care domains for mentally ill patients. The case report aims to endorse integrating both models into the management of the psychiatric population to promote patient-centered and optimal quality of care.

## Introduction

Schizophrenia is a severe psychiatric condition with an estimated lifetime prevalence of 1% in the general population [1]. The disease has contributory factors stemming from genetic and environmental aspects, with experts shifting investigative focus between epigenetics, pleiotropy, and endophenotype. The condition induces disabling behaviors such as disorganized thought, hallucinations, delusions, apathy, paranoia, and incoherent speech [2]. Disease management becomes even more challenging when paired with comorbidities such as cardiovascular and respiratory issues. Various studies have proposed that schizophrenia predisposes individuals to a higher risk of cardiovascular complications due to increased platelet reactivity, autonomic nervous system dysfunction, and antipsychotic medication treatment leading to hypertension and abnormal lipid levels [3,4]. In addition, chronic psychosis patients tend to have decreased lung function and increased risks of chronic bronchitis, chronic obstructive pulmonary disease, and pneumonia [5]. The multifactorial nature of the disease pushes for an interdisciplinary approach, as the current model fails to effectively manage patients with schizophrenia, putting a considerable burden on society through inefficient utilization of health care and exhaustion of social services [6].

The holistic model in healthcare primarily focuses on the patient being at the center of care, with treatment oriented toward managing the entire person instead of solely presenting symptoms. Researchers and healthcare professionals have further clarified this notion by advocating consideration of patients' societal, spiritual, psychological, and physiological aspects in their medical management. In this paper, the standard for holistic care will address individuals' spiritual, emotional, psychological, and physiologic health [7]. The holistic approach is especially applicable to the mentally ill patient population, as these individuals' psychological and emotional impairments necessitate high-quality care to decrease social costs.

In comparison, the Institute of Medicine proposes six domains in the quality of the patient care model when assessing and implementing health policies: safety, effectiveness, patient-centeredness, timeliness, efficiency, and equity [8]. Due to factors pertaining to their care, attaining ideal results in mentioned domains with mentally ill patient populations are challenging. Consequently, the quality of care for psychiatric patients is suboptimal compared to the general population.

In terms of current literature, there has been thus far limited publication assessing various models for managing psychiatric care. The holistic approach, for example, has been a buzzword in the medical community, with tons of paper outlining it as the model for delivering care, yet few investigations have been done in the setting of mentally ill care. Although some experts can argue that psychiatric care is synonymous with holistic care, there remains a need for definitive guidelines regarding what curtails a holistic approach to psychiatric patients. Similarly, the quality care model has been absent from scientific investigations in psychiatric care, posing whether the quality care standard should be strived for in the psychiatric population.

This paper will look closely at a middle-aged white male patient with schizophrenia and cardiopulmonary concerns that arose during his hospital course, analyzing the patient's quality of care. The aim is to expand on the discussion of offering the vulnerable mentally ill patient holistic care with healthcare quality standards and propose approaches integrating both practices into future models of care.

## Case presentation

Mr. R was a 59-year-old white male with a medical history of schizophrenia. Chronically homeless, the patient was found trying to cut out his prolapsed rectum that, had persisted for about six months by a psychiatric outreach team and transferred to the psychiatric emergency department. During the initial psychiatric evaluation, the patient displayed guarded behavior toward hospital staff, speaking in a monotone voice, responding to internal stimuli, and being unaware of his schizophrenic condition. Mr. R was also afebrile with normal vitals at the initial presentation to the Emergency department. During the physical exam, he was only found to be significant for prolapsed rectum measuring in length 2.5 inches with no sign of ulceration but reporting regular underwear change due to persistent bloody stool prior to hospitalization. Mr. R's initial labs showed low hemoglobin levels of 7.8 g/dL, indicating anemia; hence, he was transferred to the medicine floor. A surgery consultation was requested to discuss the possible reduction of the prolapsed rectum, as his anemia was likely due to blood loss secondary to his prolapsed rectum. Mr. R was educated on his condition and offered surgical and blood transfusion intervention, though he refused both. Two separate capacity evaluations found that Mr. R had the medical decision-making capacity to refuse these interventions. Mr. R consented to the oral iron supplements to raise his hemoglobin, and shortly after, his levels became stable enough to be transferred from Medicine to the inpatient psychiatry service.

In the psychiatric unit, Mr. R initially refused to take oral antipsychotic medication. After discussing the risks and benefits, the patient agreed to take Olanzapine 5 mg twice daily. Later, the patient transitioned to oral Risperidone due to minimal improvement in alleviating the voices he was hearing or diminished expressive and vocal gestures. He was eventually switched to Paliperidone Palmitate Extended-Release Injectable Suspension 234 mg with a last loading dose of 156mg seven days later. After several weeks with the new antipsychotic regimen, the patient's ongoing auditory hallucinations dramatically reduced. He began demonstrating emotions and behaviors such as laughter, jokes, and frustration due to his prolonged stay, indicating signs of improvement in his psychosis symptoms.

Mr. R was an individual who took great pride in his independence, stating that he had survived homelessness alone since 1976. Mr. R was visited by his care team every morning, and during each encounter was offered help with changing bed linens, fetching his belongings, or helping him walk to his wheelchair. Mr. R refused assistance almost every time and would push himself to show staff his capacity to perform activities of daily living. Unfortunately, due to Mr. R's schizophrenia and a regional COVID-19 outbreak, his placement was delayed as it was difficult for the care team to find a suitable facility to manage his needs. As a result, his stay in the hospital was prolonged by several weeks, instilling feelings of hopelessness and frustration. Mr. R began to isolate himself and became socially withdrawn, unkempt, disheveled, and depressed as time passed in the hospital with no placement available.

As a result of being confined to the medicine unit for three days and then the psych unit for fifty days, gradually, Mr. R became unmotivated, socially withdrawn, and depressed due to limited autonomy. Thus, he became less motivated to shower, eat adequately, converse with others, walk around the unit, and sleep enough. Over weeks, this effect accumulated and contributed to the patient's deconditioning. Toward his last weeks in the hospital before discharge, the patient endorsed new-onset shortness of breath and general weakness. Mr. R denied any symptoms of chest pain, pain on inspiration, radiating pain, or leg pain. The patient, already becoming increasingly apathetic due to his prolonged hospital course and loss of autonomy, lost further drive with these new cardiopulmonary symptoms.

The psychiatric team decided to consult the Medicine team, followed by an extensive workup, including a negative chest x-ray, complete chemistry and hematology panel with no significant abnormalities, troponin level less than 0.010 ng/mL, and normal EKG with no sign of arrhythmia or ischemia. The patient also had a chest CT scan, where he was given sedative medication due to his fears of radiation. At the end of the workup, no clear cardiopulmonary etiology became evident. Medicine and Psychiatry concluded that the patient's shortness of breath was most likely due to physical deconditioning. 

With no findings after a thorough workup of his shortness of breath, Mr. R became more distraught and depressed, believing that his body was failing and that he would die soon in the hospital. Hence, to improve Mr. R's mood, the care team incorporated interactive and stimulating activities into his daily schedule. In the following days, team members would meet with him on separate occasions to discuss his interests, such as discussing the books he had kept over the years when he was on the streets. The team worked with Mr. R to ensure he could eat the food he enjoyed uplifting his mood. He was encouraged to participate in group activities and walk around the psychiatric floor with the physical therapist. In addition, whenever the patient demonstrated social withdrawal or apathy, he was reminded by the care team of his life journey and how he had survived on his own for such a long time. These activities and exercises uplifted Mr. R's spirit, and he willingly regained his motivation to participate in the ward. In addition, the psychiatric team recommended in Mr. R's discharge packet to implement similar ward activities to improve his stay at the skilled nursing facility.

## Discussion

Patients with a history of schizophrenia can present to healthcare facilities with various medical complaints and significant positive, cognitive, and negative psychosis symptoms. Mr. R's case presents a chronically unhoused patient with schizophrenia who survived on the street for decades and became hospitalized due to attempts to treat his rectal prolapse by himself. The patient's lack of psychiatric treatment led to his inability to promptly seek appropriate care for his prolapse rectum. Consequently, he allowed the condition to exacerbate further and cause anemia along with symptoms of fatigue and weakness. Mr. R presented to the hospital at an advanced stage of his disease pathology, which is significantly more costly to the healthcare system and utilizes more healthcare resources, preventing the quality-of-care model from achieving efficiency of care.

Mr. R's sudden shortness of breath led to an extensive workup, including a chest x-ray, D-dimer, CT angiogram, troponin levels, labs, and EKG, all unremarkable. The listed diagnostic tests' objective was to primarily rule out severe disease conditions such as pulmonary embolism, myocardial ischemia, and pneumothorax. Ultimately, the psychiatry and medicine teams reasoned that the patient's symptoms stemmed from deconditioning. The etiology of deconditioning was perceived to have gradually arisen from the patient's ongoing deteriorating mental state. A regional COVID-19 outbreak delayed Mr. R's placement, instilling in him feelings of depression and hopelessness. The delay in Mr. R's placement caused significant harm both in his psychological and emotional health, which he coped with through increased bedrest, physical inactivity, reduced social interaction, and lack of appetite. In this case, shortcomings in achieving optimal patient-centered care stemmed from failing to holistically approach the patient and consider their psychological and emotional needs. As a result, the treatment course was prolonged and complicated, falling short of meeting the ideal standard for care effectiveness and efficiency outlined by the quality care model.

Patients with severe mental illnesses such as schizophrenia have high sensitivity to stress and are prone to misinterpretation of information [9]. Furthermore, schizophrenia's symptomology of disorganized thought hampers a patient's communication ability, making them more reliant on physicians to sufficiently discuss the diagnostic purpose of further steps in the treatment course [10]. Therefore, physicians must utilize a care model that holistically approaches the mentally ill patient, analyzing the individual as a whole and avoiding missing any of their needs in conjunction with quality care metrics to ensure both delivery and result of care are optimal. This topic is worthy of further investigation, with the potential to significantly improve psychiatric care and reduce healthcare and societal expenditure.

## Conclusions

This paper highlights potential pitfalls when managing mentally ill patients and possible approaches to further improving care and cost, which is worth investigating in future literature. Mr. R, with a history of schizophrenia due to his disabling symptoms, had poor cognition to communicate with his healthcare team his needs and concerns. The patient's emotional and psychological state throughout his hospital stays deteriorated due to delayed facility placement and an unforeseeable regional COVID-19 outbreak, leading to the patient's deconditioning. Mr. R's hospital course became further complicated by a sudden onset of shortness of breath symptoms secondary to deconditioning, and the symptoms were investigated extensively for a cardiopulmonary etiology. After ruling out cardiopulmonary etiologies, the care team focused on improving the patient's mood and drive through dietary modification, physical therapy sessions, and social interaction. These measures significantly improved Mr. R's emotional and psychological state before discharge with a plan of care to follow outpatient.

This case signifies the importance of utilizing quality care and holistic models to evaluate patients and plan care. By doing so, medical therapy can better treat a patient's underlying causes to manage symptoms and promote high-end care. Consequently, patient care for psychiatric patients can become efficient and effective and maintain patient-centeredness by guiding therapies toward meeting psychological and emotional needs.
